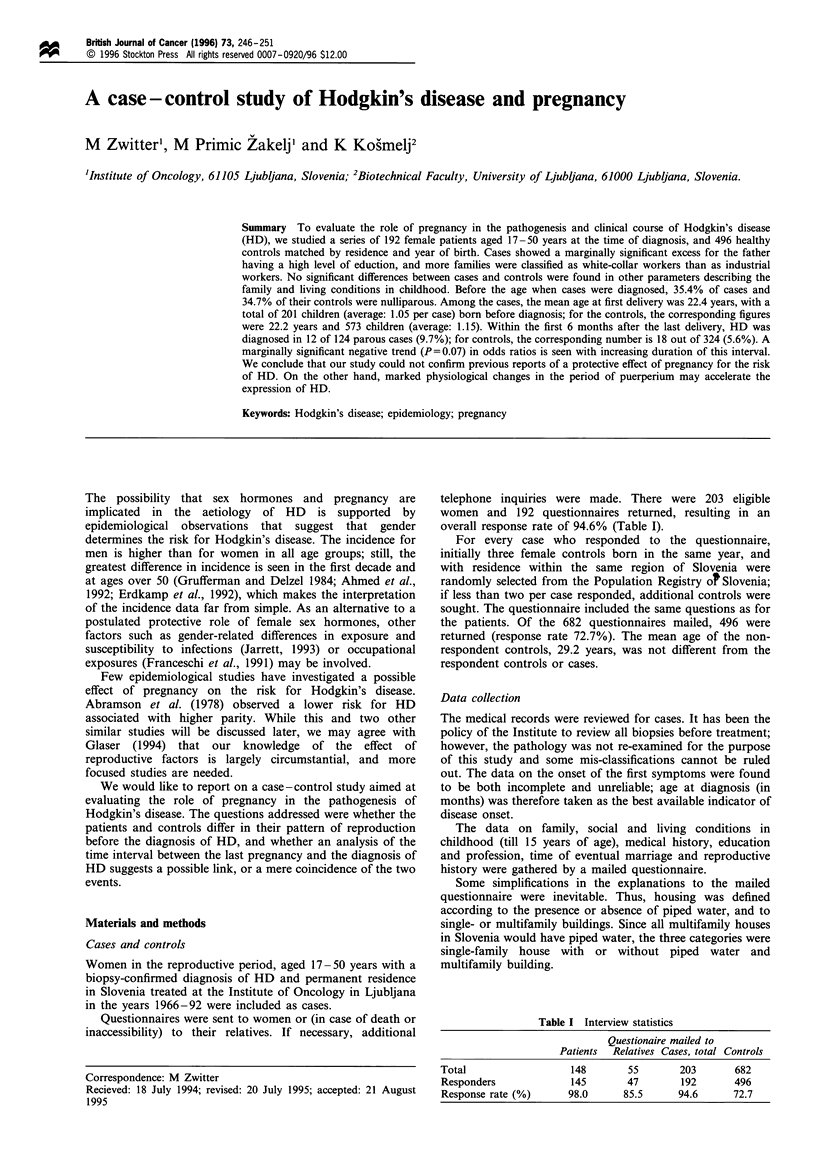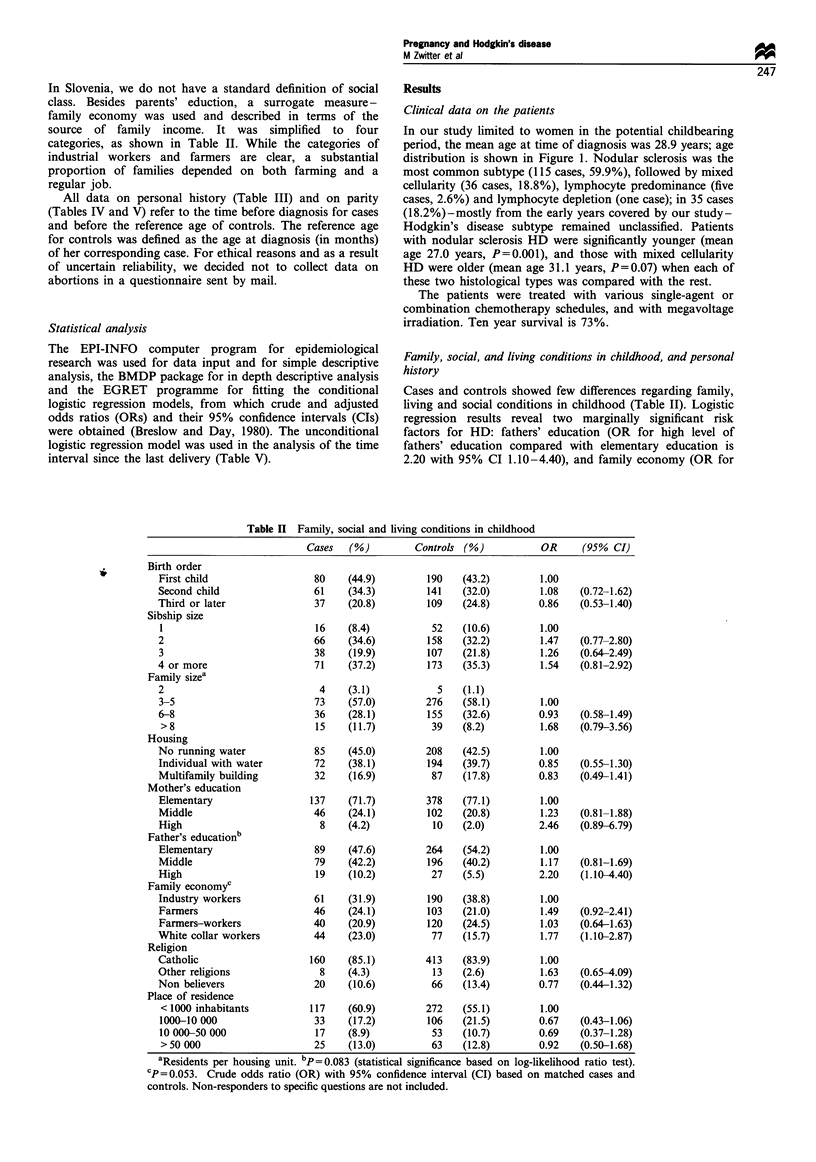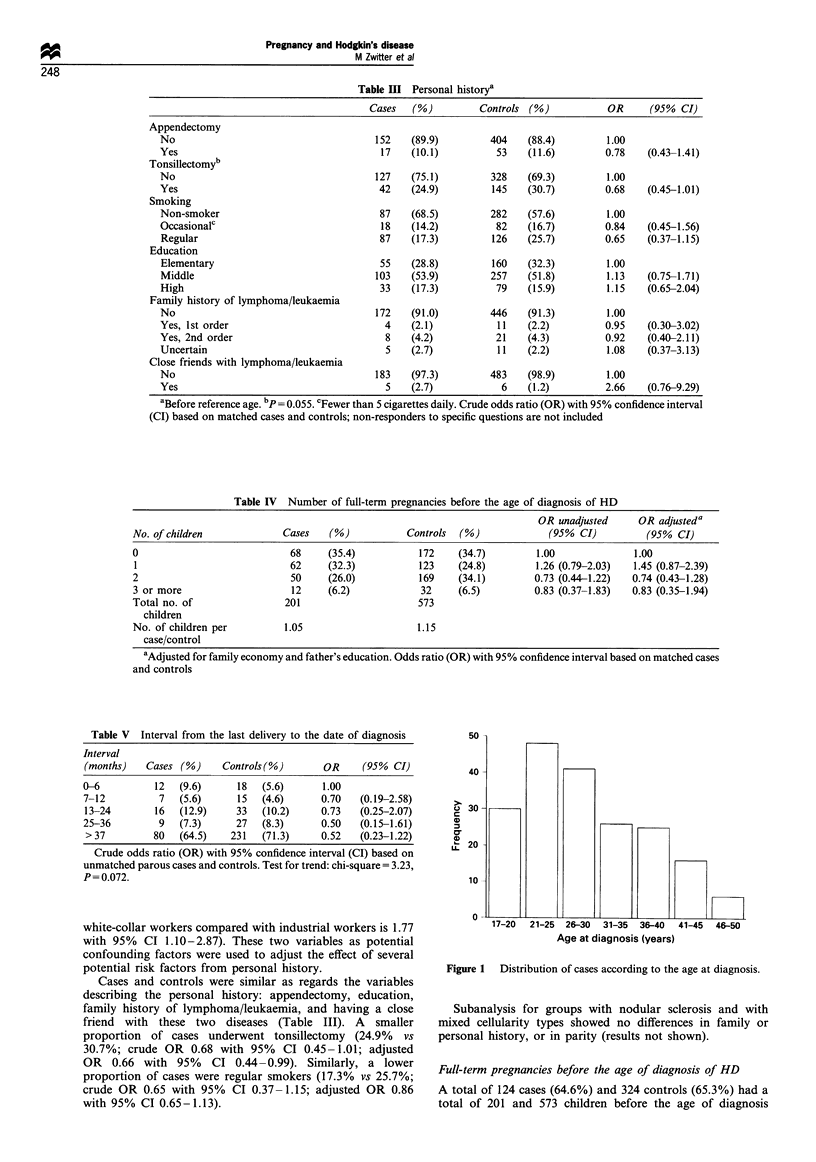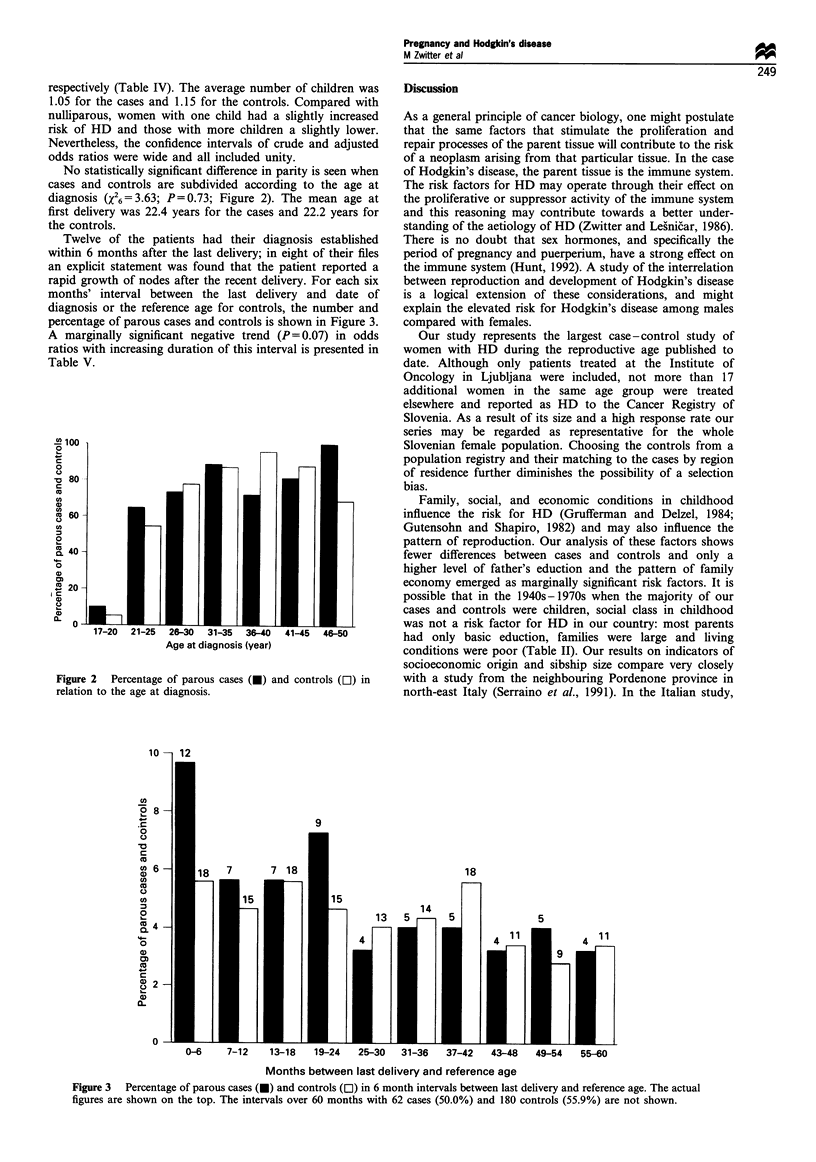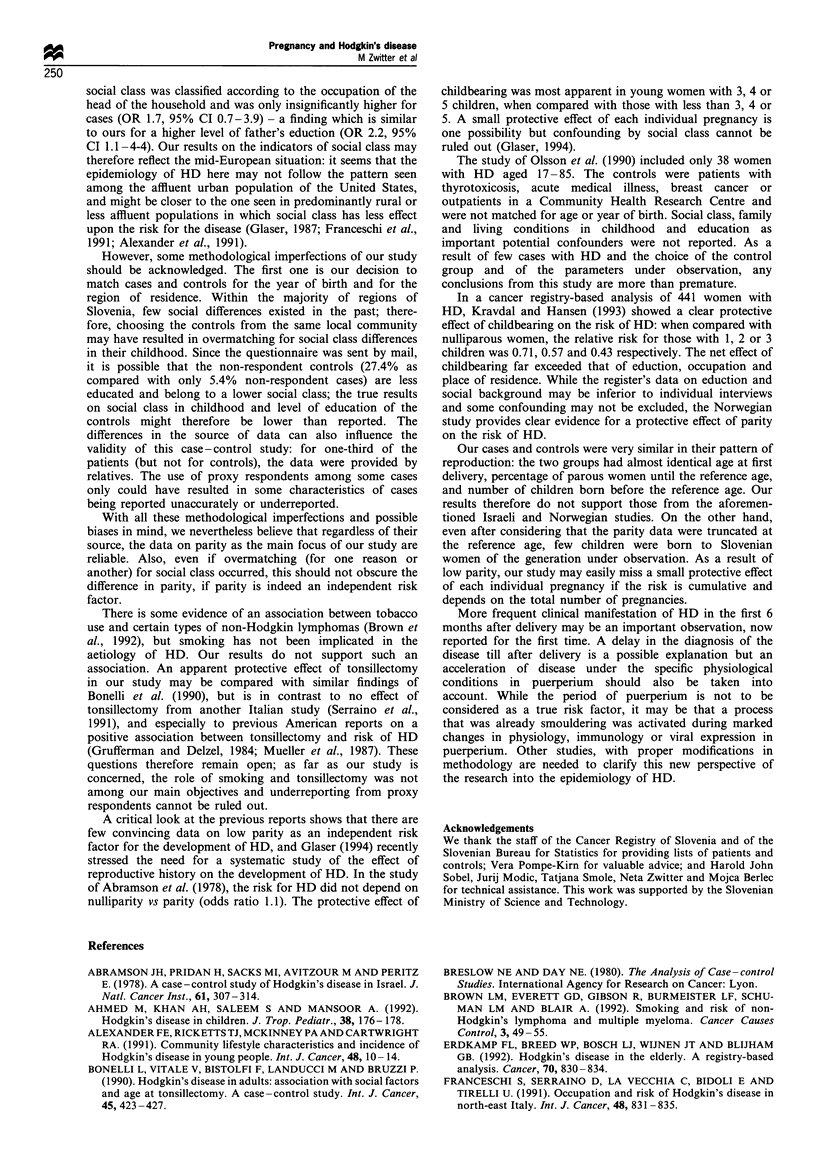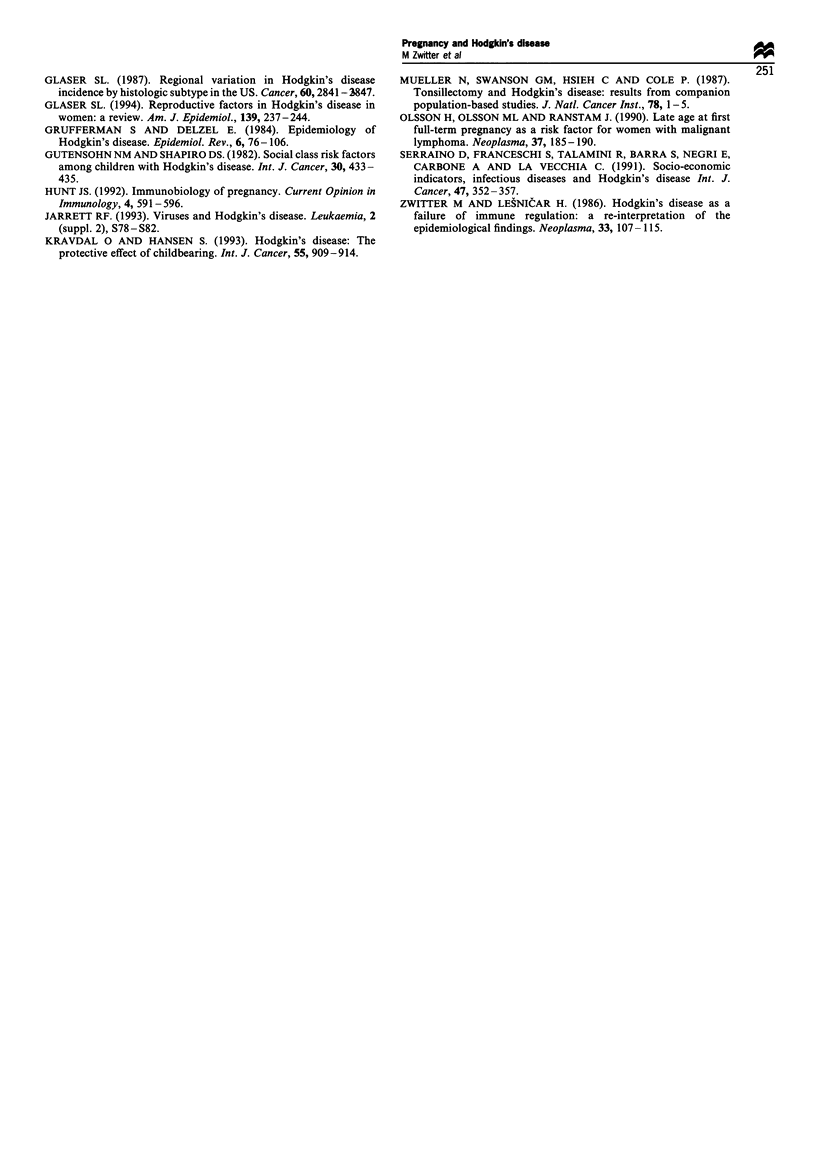# A case-control study of Hodgkin's disease and pregnancy.

**DOI:** 10.1038/bjc.1996.43

**Published:** 1996-01

**Authors:** M. Zwitter, M. P. Zakelj, K. Kosmelj

**Affiliations:** Institute of Oncology, Ljubljana, Slovenia.

## Abstract

To evaluate the role of pregnancy in the pathogenesis and clinical course of Hodgkin's disease (HD), we studied a series of 192 female patients aged 17-50 years at the time of diagnosis, and 496 healthy controls matched by residence and year of birth. Cases showed a marginally significant excess for the father having a high level of education, and more families were classified as white-collar workers than as industrial workers. No significant differences between cases and controls were found in other parameters describing the family and living conditions in childhood. Before the age when cases were diagnosed, 35.4% of cases and 34.7% of their controls were nulliparous. Among the cases, the mean age at first delivery was 22.4 years, with a total of 201 children (average: 1.05 per case) born before diagnosis; for the controls, the corresponding figures were 22.2 years and 573 children (average: 1.15). Within the first 6 months after the last delivery, HD was diagnosed in 12 of 124 parous cases (9.7%); for controls, the corresponding number is 18 out of 324 (5.6%). A marginally significant negative trend (P = 0.07) in odds ratios is seen with increasing duration of this interval. We conclude that our study could not confirm previous reports of a protective effect of pregnancy for the risk of HD. On the other hand, marked physiological changes in the period of puerperium may accelerate the expression of HD.


British Journal of Cancer (1996) 73, 246-251
© 1996 Stockton Press All rights reserved 0007-0920/96 $12.00

### A case-control study of Hodgkin's disease and pregnancy

M Zwitter[1], M Primic Žakelj[1] and K Košmelj[2]

[1]Institute of Oncology, 61105 Ljubljana, Slovenia; [2]Biotechnical Faculty, University of Ljubljana, 61000 Ljubljana, Slovenia.

**Summary** To evaluate the role of pregnancy in the pathogenesis and clinical course of Hodgkin's disease (HD), we studied a series of 192 female patients aged 17-50 years at the time of diagnosis, and 496 healthy controls matched by residence and year of birth. Cases showed a marginally significant excess for the father having a high level of eduction, and more families were classified as white-collar workers than as industrial workers. No significant differences between cases and controls were found in other parameters describing the family and living conditions in childhood. Before the age when cases were diagnosed, 35.4% of cases and 34.7% of their controls were nulliparous. Among the cases, the mean age at first delivery was 22.4 years, with a total of 201 children (average: 1.05 per case) born before diagnosis; for the controls, the corresponding figures were 22.2 years and 573 children (average: 1.15). Within the first 6 months after the last delivery, HD was diagnosed in 12 of 124 parous cases (9.7%); for controls, the corresponding number is 18 out of 324 (5.6%). A marginally significant negative trend ($P=0.07$) in odds ratios is seen with increasing duration of this interval. We conclude that our study could not confirm previous reports of a protective effect of pregnancy for the risk of HD. On the other hand, marked physiological changes in the period of puerperium may accelerate the expression of HD.

**Keywords:** Hodgkin's disease; epidemiology; pregnancy

The possibility that sex hormones and pregnancy are implicated in the aetiology of HD is supported by epidemiological observations that suggest that gender determines the risk for Hodgkin's disease. The incidence for men is higher than for women in all age groups; still, the greatest difference in incidence is seen in the first decade and at ages over 50 (Grufferman and Delzel 1984; Ahmed *et al.*, 1992; Erdkamp *et al.*, 1992), which makes the interpretation of the incidence data far from simple. As an alternative to a postulated protective role of female sex hormones, other factors such as gender-related differences in exposure and susceptibility to infections (Jarrett, 1993) or occupational exposures (Franceschi *et al.*, 1991) may be involved.

Few epidemiological studies have investigated a possible effect of pregnancy on the risk for Hodgkin's disease. Abramson *et al.* (1978) observed a lower risk for HD associated with higher parity. While this and two other similar studies will be discussed later, we may agree with Glaser (1994) that our knowledge of the effect of reproductive factors is largely circumstantial, and more focused studies are needed.

We would like to report on a case-control study aimed at evaluating the role of pregnancy in the pathogenesis of Hodgkin's disease. The questions addressed were whether the patients and controls differ in their pattern of reproduction before the diagnosis of HD, and whether an analysis of the time interval between the last pregnancy and the diagnosis of HD suggests a possible link, or a mere coincidence of the two events.

### Materials and methods

### *Cases and controls*

Women in the reproductive period, aged 17-50 years with a biopsy-confirmed diagnosis of HD and permanent residence in Slovenia treated at the Institute of Oncology in Ljubljana in the years 1966-92 were included as cases.

Questionnaires were sent to women or (in case of death or inaccessibility) to their relatives. If necessary, additional telephone inquiries were made. There were 203 eligible women and 192 questionnaires returned, resulting in an overall response rate of 94.6% (Table I).

For every case who responded to the questionnaire, initially three female controls born in the same year, and with residence within the same region of Slovenia were randomly selected from the Population Registry of Slovenia; if less than two per case responded, additional controls were sought. The questionnaire included the same questions as for the patients. Of the 682 questionnaires mailed, 496 were returned (response rate 72.7%). The mean age of the non-respondent controls, 29.2 years, was not different from the respondent controls or cases.

### *Data collection*

The medical records were reviewed for cases. It has been the policy of the Institute to review all biopsies before treatment; however, the pathology was not re-examined for the purpose of this study and some mis-classifications cannot be ruled out. The data on the onset of the first symptoms were found to be both incomplete and unreliable; age at diagnosis (in months) was therefore taken as the best available indicator of disease onset.

The data on family, social and living conditions in childhood (till 15 years of age), medical history, education and profession, time of eventual marriage and reproductive history were gathered by a mailed questionnaire.

Some simplifications in the explanations to the mailed questionnaire were inevitable. Thus, housing was defined according to the presence or absence of piped water, and to single- or multifamily buildings. Since all multifamily houses in Slovenia would have piped water, the three categories were single-family house with or without piped water and multifamily building.

**Table I** Interview statistics

| | | *Questionaire mailed to* | | |
|---|---|---|---|---|
| | *Patients* | *Relatives* | *Cases, total* | *Controls* |
| Total | 148 | 55 | 203 | 682 |
| Responders | 145 | 47 | 192 | 496 |
| Response rate (%) | 98.0 | 85.5 | 94.6 | 72.7 |

Correspondence: M Zwitter
Recieved: 18 July 1994; revised: 20 July 1995; accepted: 21 August 1995

In Slovenia, we do not have a standard definition of social class. Besides parents' eduction, a surrogate measure–family economy was used and described in terms of the source of family income. It was simplified to four categories, as shown in Table II. While the categories of industrial workers and farmers are clear, a substantial proportion of families depended on both farming and a regular job.

All data on personal history (Table III) and on parity (Tables IV and V) refer to the time before diagnosis for cases and before the reference age of controls. The reference age for controls was defined as the age at diagnosis (in months) of her corresponding case. For ethical reasons and as a result of uncertain reliability, we decided not to collect data on abortions in a questionnaire sent by mail.

### *Statistical analysis*

The EPI-INFO computer program for epidemiological research was used for data input and for simple descriptive analysis, the BMDP package for in depth descriptive analysis and the EGRET programme for fitting the conditional logistic regression models, from which crude and adjusted odds ratios (ORs) and their 95% confidence intervals (CIs) were obtained (Breslow and Day, 1980). The unconditional logistic regression model was used in the analysis of the time interval since the last delivery (Table V).

### Results

### *Clinical data on the patients*

In our study limited to women in the potential childbearing period, the mean age at time of diagnosis was 28.9 years; age distribution is shown in Figure 1. Nodular sclerosis was the most common subtype (115 cases, 59.9%), followed by mixed cellularity (36 cases, 18.8%), lymphocyte predominance (five cases, 2.6%) and lymphocyte depletion (one case); in 35 cases (18.2%)–mostly from the early years covered by our study–Hodgkin's disease subtype remained unclassified. Patients with nodular sclerosis HD were significantly younger (mean age 27.0 years, $P=0.001$), and those with mixed cellularity HD were older (mean age 31.1 years, $P=0.07$) when each of these two histological types was compared with the rest.

The patients were treated with various single-agent or combination chemotherapy schedules, and with megavoltage irradiation. Ten year survival is 73%.

### *Family, social, and living conditions in childhood, and personal history*

Cases and controls showed few differences regarding family, living and social conditions in childhood (Table II). Logistic regression results reveal two marginally significant risk factors for HD: fathers' education (OR for high level of fathers' education compared with elementary education is 2.20 with 95% CI 1.10–4.40), and family economy (OR for

**Table II** Family, social and living conditions in childhood

| | *Cases* | *(%)* | *Controls* | *(%)* | *OR* | *(95% CI)* |
|---|---|---|---|---|---|---|
| Birth order | | | | | | |
| First child | 80 | (44.9) | 190 | (43.2) | 1.00 | |
| Second child | 61 | (34.3) | 141 | (32.0) | 1.08 | (0.72–1.62) |
| Third or later | 37 | (20.8) | 109 | (24.8) | 0.86 | (0.53–1.40) |
| Sibship size | | | | | | |
| 1 | 16 | (8.4) | 52 | (10.6) | 1.00 | |
| 2 | 66 | (34.6) | 158 | (32.2) | 1.47 | (0.77–2.80) |
| 3 | 38 | (19.9) | 107 | (21.8) | 1.26 | (0.64–2.49) |
| 4 or more | 71 | (37.2) | 173 | (35.3) | 1.54 | (0.81–2.92) |
| Family size[a] | | | | | | |
| 2 | 4 | (3.1) | 5 | (1.1) | | |
| 3–5 | 73 | (57.0) | 276 | (58.1) | 1.00 | |
| 6–8 | 36 | (28.1) | 155 | (32.6) | 0.93 | (0.58–1.49) |
| >8 | 15 | (11.7) | 39 | (8.2) | 1.68 | (0.79–3.56) |
| Housing | | | | | | |
| No running water | 85 | (45.0) | 208 | (42.5) | 1.00 | |
| Individual with water | 72 | (38.1) | 194 | (39.7) | 0.85 | (0.55–1.30) |
| Multifamily building | 32 | (16.9) | 87 | (17.8) | 0.83 | (0.49–1.41) |
| Mother's education | | | | | | |
| Elementary | 137 | (71.7) | 378 | (77.1) | 1.00 | |
| Middle | 46 | (24.1) | 102 | (20.8) | 1.23 | (0.81–1.88) |
| High | 8 | (4.2) | 10 | (2.0) | 2.46 | (0.89–6.79) |
| Father's education[b] | | | | | | |
| Elementary | 89 | (47.6) | 264 | (54.2) | 1.00 | |
| Middle | 79 | (42.2) | 196 | (40.2) | 1.17 | (0.81–1.69) |
| High | 19 | (10.2) | 27 | (5.5) | 2.20 | (1.10–4.40) |
| Family economy[c] | | | | | | |
| Industry workers | 61 | (31.9) | 190 | (38.8) | 1.00 | |
| Farmers | 46 | (24.1) | 103 | (21.0) | 1.49 | (0.92–2.41) |
| Farmers–workers | 40 | (20.9) | 120 | (24.5) | 1.03 | (0.64–1.63) |
| White collar workers | 44 | (23.0) | 77 | (15.7) | 1.77 | (1.10–2.87) |
| Religion | | | | | | |
| Catholic | 160 | (85.1) | 413 | (83.9) | 1.00 | |
| Other religions | 8 | (4.3) | 13 | (2.6) | 1.63 | (0.65–4.09) |
| Non believers | 20 | (10.6) | 66 | (13.4) | 0.77 | (0.44–1.32) |
| Place of residence | | | | | | |
| <1000 inhabitants | 117 | (60.9) | 272 | (55.1) | 1.00 | |
| 1000–10 000 | 33 | (17.2) | 106 | (21.5) | 0.67 | (0.43–1.06) |
| 10 000–50 000 | 17 | (8.9) | 53 | (10.7) | 0.69 | (0.37–1.28) |
| >50 000 | 25 | (13.0) | 63 | (12.8) | 0.92 | (0.50–1.68) |

[a]Residents per housing unit. [b]$P=0.083$ (statistical significance based on log-likelihood ratio test). [c]$P=0.053$. Crude odds ratio (OR) with 95% confidence interval (CI) based on matched cases and controls. Non-responders to specific questions are not included.

**Table III** Personal history[a]

| | Cases | (%) | Controls | (%) | OR | (95% CI) |
|---|---|---|---|---|---|---|
| Appendectomy | | | | | | |
| No | 152 | (89.9) | 404 | (88.4) | 1.00 | |
| Yes | 17 | (10.1) | 53 | (11.6) | 0.78 | (0.43–1.41) |
| Tonsillectomy[b] | | | | | | |
| No | 127 | (75.1) | 328 | (69.3) | 1.00 | |
| Yes | 42 | (24.9) | 145 | (30.7) | 0.68 | (0.45–1.01) |
| Smoking | | | | | | |
| Non-smoker | 87 | (68.5) | 282 | (57.6) | 1.00 | |
| Occasional[c] | 18 | (14.2) | 82 | (16.7) | 0.84 | (0.45–1.56) |
| Regular | 87 | (17.3) | 126 | (25.7) | 0.65 | (0.37–1.15) |
| Education | | | | | | |
| Elementary | 55 | (28.8) | 160 | (32.3) | 1.00 | |
| Middle | 103 | (53.9) | 257 | (51.8) | 1.13 | (0.75–1.71) |
| High | 33 | (17.3) | 79 | (15.9) | 1.15 | (0.65–2.04) |
| Family history of lymphoma/leukaemia | | | | | | |
| No | 172 | (91.0) | 446 | (91.3) | 1.00 | |
| Yes, 1st order | 4 | (2.1) | 11 | (2.2) | 0.95 | (0.30–3.02) |
| Yes, 2nd order | 8 | (4.2) | 21 | (4.3) | 0.92 | (0.40–2.11) |
| Uncertain | 5 | (2.7) | 11 | (2.2) | 1.08 | (0.37–3.13) |
| Close friends with lymphoma/leukaemia | | | | | | |
| No | 183 | (97.3) | 483 | (98.9) | 1.00 | |
| Yes | 5 | (2.7) | 6 | (1.2) | 2.66 | (0.76–9.29) |

[a]Before reference age. [b]$P=0.055$. [c]Fewer than 5 cigarettes daily. Crude odds ratio (OR) with 95% confidence interval (CI) based on matched cases and controls; non-responders to specific questions are not included

**Table IV** Number of full-term pregnancies before the age of diagnosis of HD

| No. of children | Cases | (%) | Controls | (%) | OR unadjusted (95% CI) | OR adjusted[a] (95% CI) |
|---|---|---|---|---|---|---|
| 0 | 68 | (35.4) | 172 | (34.7) | 1.00 | 1.00 |
| 1 | 62 | (32.3) | 123 | (24.8) | 1.26 (0.79–2.03) | 1.45 (0.87–2.39) |
| 2 | 50 | (26.0) | 169 | (34.1) | 0.73 (0.44–1.22) | 0.74 (0.43–1.28) |
| 3 or more | 12 | (6.2) | 32 | (6.5) | 0.83 (0.37–1.83) | 0.83 (0.35–1.94) |
| Total no. of children | 201 | | 573 | | | |
| No. of children per case/control | 1.05 | | 1.15 | | | |

[a]Adjusted for family economy and father's education. Odds ratio (OR) with 95% confidence interval based on matched cases and controls

**Table V** Interval from the last delivery to the date of diagnosis

| Interval (months) | Cases | (%) | Controls | (%) | OR | (95% CI) |
|---|---|---|---|---|---|---|
| 0–6 | 12 | (9.6) | 18 | (5.6) | 1.00 | |
| 7–12 | 7 | (5.6) | 15 | (4.6) | 0.70 | (0.19–2.58) |
| 13–24 | 16 | (12.9) | 33 | (10.2) | 0.73 | (0.25–2.07) |
| 25–36 | 9 | (7.3) | 27 | (8.3) | 0.50 | (0.15–1.61) |
| >37 | 80 | (64.5) | 231 | (71.3) | 0.52 | (0.23–1.22) |

Crude odds ratio (OR) with 95% confidence interval (CI) based on unmatched parous cases and controls. Test for trend: chi-square = 3.23, $P=0.072$.

white-collar workers compared with industrial workers is 1.77 with 95% CI 1.10–2.87). These two variables as potential confounding factors were used to adjust the effect of several potential risk factors from personal history.

Cases and controls were similar as regards the variables describing the personal history: appendectomy, education, family history of lymphoma/leukaemia, and having a close friend with these two diseases (Table III). A smaller proportion of cases underwent tonsillectomy (24.9% *vs* 30.7%; crude OR 0.68 with 95% CI 0.45–1.01; adjusted OR 0.66 with 95% CI 0.44–0.99). Similarly, a lower proportion of cases were regular smokers (17.3% *vs* 25.7%; crude OR 0.65 with 95% CI 0.37–1.15; adjusted OR 0.86 with 95% CI 0.65–1.13).

**Figure 1** Distribution of cases according to the age at diagnosis.

Subanalysis for groups with nodular sclerosis and with mixed cellularity types showed no differences in family or personal history, or in parity (results not shown).

*Full-term pregnancies before the age of diagnosis of HD*

A total of 124 cases (64.6%) and 324 controls (65.3%) had a total of 201 and 573 children before the age of diagnosis respectively (Table IV). The average number of children was 1.05 for the cases and 1.15 for the controls. Compared with nulliparous, women with one child had a slightly increased risk of HD and those with more children a slightly lower. Nevertheless, the confidence intervals of crude and adjusted odds ratios were wide and all included unity.

No statistically significant difference in parity is seen when cases and controls are subdivided according to the age at diagnosis ($\chi^2_6 = 3.63$; $P = 0.73$; Figure 2). The mean age at first delivery was 22.4 years for the cases and 22.2 years for the controls.

Twelve of the patients had their diagnosis established within 6 months after the last delivery; in eight of their files an explicit statement was found that the patient reported a rapid growth of nodes after the recent delivery. For each six months' interval between the last delivery and date of diagnosis or the reference age for controls, the number and percentage of parous cases and controls is shown in Figure 3. A marginally significant negative trend ($P = 0.07$) in odds ratios with increasing duration of this interval is presented in Table V.

**Figure 2** Percentage of parous cases (■) and controls (□) in relation to the age at diagnosis.

### Discussion

As a general principle of cancer biology, one might postulate that the same factors that stimulate the proliferation and repair processes of the parent tissue will contribute to the risk of a neoplasm arising from that particular tissue. In the case of Hodgkin's disease, the parent tissue is the immune system. The risk factors for HD may operate through their effect on the proliferative or suppressor activity of the immune system and this reasoning may contribute towards a better understanding of the aetiology of HD (Zwitter and Lešničar, 1986). There is no doubt that sex hormones, and specifically the period of pregnancy and puerperium, have a strong effect on the immune system (Hunt, 1992). A study of the interrelation between reproduction and development of Hodgkin's disease is a logical extension of these considerations, and might explain the elevated risk for Hodgkin's disease among males compared with females.

Our study represents the largest case–control study of women with HD during the reproductive age published to date. Although only patients treated at the Institute of Oncology in Ljubljana were included, not more than 17 additional women in the same age group were treated elsewhere and reported as HD to the Cancer Registry of Slovenia. As a result of its size and a high response rate our series may be regarded as representative for the whole Slovenian female population. Choosing the controls from a population registry and their matching to the cases by region of residence further diminishes the possibility of a selection bias.

Family, social, and economic conditions in childhood influence the risk for HD (Grufferman and Delzel, 1984; Gutensohn and Shapiro, 1982) and may also influence the pattern of reproduction. Our analysis of these factors shows fewer differences between cases and controls and only a higher level of father's eduction and the pattern of family economy emerged as marginally significant risk factors. It is possible that in the 1940s–1970s when the majority of our cases and controls were children, social class in childhood was not a risk factor for HD in our country: most parents had only basic eduction, families were large and living conditions were poor (Table II). Our results on indicators of socioeconomic origin and sibship size compare very closely with a study from the neighbouring Pordenone province in north-east Italy (Serraino *et al.*, 1991). In the Italian study,

**Figure 3** Percentage of parous cases (■) and controls (□) in 6 month intervals between last delivery and reference age. The actual figures are shown on the top. The intervals over 60 months with 62 cases (50.0%) and 180 controls (55.9%) are not shown.

social class was classified according to the occupation of the head of the household and was only insignificantly higher for cases (OR 1.7, 95% CI 0.7–3.9) – a finding which is similar to ours for a higher level of father's eduction (OR 2.2, 95% CI 1.1–4-4). Our results on the indicators of social class may therefore reflect the mid-European situation: it seems that the epidemiology of HD here may not follow the pattern seen among the affluent urban population of the United States, and might be closer to the one seen in predominantly rural or less affluent populations in which social class has less effect upon the risk for the disease (Glaser, 1987; Franceschi *et al.*, 1991; Alexander *et al.*, 1991).

However, some methodological imperfections of our study should be acknowledged. The first one is our decision to match cases and controls for the year of birth and for the region of residence. Within the majority of regions of Slovenia, few social differences existed in the past; therefore, choosing the controls from the same local community may have resulted in overmatching for social class differences in their childhood. Since the questionnaire was sent by mail, it is possible that the non-respondent controls (27.4% as compared with only 5.4% non-respondent cases) are less educated and belong to a lower social class; the true results on social class in childhood and level of education of the controls might therefore be lower than reported. The differences in the source of data can also influence the validity of this case–control study: for one-third of the patients (but not for controls), the data were provided by relatives. The use of proxy respondents among some cases only could have resulted in some characteristics of cases being reported unaccurately or underreported.

With all these methodological imperfections and possible biases in mind, we nevertheless believe that regardless of their source, the data on parity as the main focus of our study are reliable. Also, even if overmatching (for one reason or another) for social class occurred, this should not obscure the difference in parity, if parity is indeed an independent risk factor.

There is some evidence of an association between tobacco use and certain types of non-Hodgkin lymphomas (Brown *et al.*, 1992), but smoking has not been implicated in the aetiology of HD. Our results do not support such an association. An apparent protective effect of tonsillectomy in our study may be compared with similar findings of Bonelli *et al.* (1990), but is in contrast to no effect of tonsillectomy from another Italian study (Serraino *et al.*, 1991), and especially to previous American reports on a positive association between tonsillectomy and risk of HD (Grufferman and Delzel, 1984; Mueller *et al.*, 1987). These questions therefore remain open; as far as our study is concerned, the role of smoking and tonsillectomy was not among our main objectives and underreporting from proxy respondents cannot be ruled out.

A critical look at the previous reports shows that there are few convincing data on low parity as an independent risk factor for the development of HD, and Glaser (1994) recently stressed the need for a systematic study of the effect of reproductive history on the development of HD. In the study of Abramson *et al.* (1978), the risk for HD did not depend on nulliparity *vs* parity (odds ratio 1.1). The protective effect of childbearing was most apparent in young women with 3, 4 or 5 children, when compared with those with less than 3, 4 or 5. A small protective effect of each individual pregnancy is one possibility but confounding by social class cannot be ruled out (Glaser, 1994).

The study of Olsson *et al.* (1990) included only 38 women with HD aged 17–85. The controls were patients with thyrotoxicosis, acute medical illness, breast cancer or outpatients in a Community Health Research Centre and were not matched for age or year of birth. Social class, family and living conditions in childhood and education as important potential confounders were not reported. As a result of few cases with HD and the choice of the control group and of the parameters under observation, any conclusions from this study are more than premature.

In a cancer registry-based analysis of 441 women with HD, Kravdal and Hansen (1993) showed a clear protective effect of childbearing on the risk of HD: when compared with nulliparous women, the relative risk for those with 1, 2 or 3 children was 0.71, 0.57 and 0.43 respectively. The net effect of childbearing far exceeded that of eduction, occupation and place of residence. While the register's data on eduction and social background may be inferior to individual interviews and some confounding may not be excluded, the Norwegian study provides clear evidence for a protective effect of parity on the risk of HD.

Our cases and controls were very similar in their pattern of reproduction: the two groups had almost identical age at first delivery, percentage of parous women until the reference age, and number of children born before the reference age. Our results therefore do not support those from the aforementioned Israeli and Norwegian studies. On the other hand, even after considering that the parity data were truncated at the reference age, few children were born to Slovenian women of the generation under observation. As a result of low parity, our study may easily miss a small protective effect of each individual pregnancy if the risk is cumulative and depends on the total number of pregnancies.

More frequent clinical manifestation of HD in the first 6 months after delivery may be an important observation, now reported for the first time. A delay in the diagnosis of the disease till after delivery is a possible explanation but an acceleration of disease under the specific physiological conditions in puerperium should also be taken into account. While the period of puerperium is not to be considered as a true risk factor, it may be that a process that was already smouldering was activated during marked changes in physiology, immunology or viral expression in puerperium. Other studies, with proper modifications in methodology are needed to clarify this new perspective of the research into the epidemiology of HD.

**Acknowledgements**
We thank the staff of the Cancer Registry of Slovenia and of the Slovenian Bureau for Statistics for providing lists of patients and controls; Vera Pompe-Kirn for valuable advice; and Harold John Sobel, Jurij Modic, Tatjana Smole, Neta Zwitter and Mojca Berlec for technical assistance. This work was supported by the Slovenian Ministry of Science and Technology.

### References

ABRAMSON JH, PRIDAN H, SACKS MI, AVITZOUR M AND PERITZ E. (1978). A case–control study of Hodgkin's disease in Israel. *J. Natl. Cancer Inst.*, **61**, 307–314.

AHMED M, KHAN AH, SALEEM S AND MANSOOR A. (1992). Hodgkin's disease in children. *J. Trop. Pediatr.*, **38**, 176–178.

ALEXANDER FE, RICKETTS TJ, MCKINNEY PA AND CARTWRIGHT RA. (1991). Community lifestyle characteristics and incidence of Hodgkin's disease in young people. *Int. J. Cancer*, **48**, 10–14.

BONELLI L, VITALE V, BISTOLFI F, LANDUCCI M AND BRUZZI P. (1990). Hodgkin's disease in adults: association with social factors and age at tonsillectomy. A case–control study. *Int. J. Cancer*, **45**, 423–427.

BRESLOW NE AND DAY NE. (1980). *The Analysis of Case–control Studies*. International Agency for Research on Cancer: Lyon.

BROWN LM, EVERETT GD, GIBSON R, BURMEISTER LF, SCHUMAN LM AND BLAIR A. (1992). Smoking and risk of non-Hodgkin's lymphoma and multiple myeloma. *Cancer Causes Control*, **3**, 49–55.

ERDKAMP FL, BREED WP, BOSCH LJ, WIJNEN JT AND BLIJHAM GB. (1992). Hodgkin's disease in the elderly. A registry-based analysis. *Cancer*, **70**, 830–834.

FRANCESCHI S, SERRAINO D, LA VECCHIA C, BIDOLI E AND TIRELLI U. (1991). Occupation and risk of Hodgkin's disease in north-east Italy. *Int. J. Cancer*, **48**, 831–835.

GLASER SL. (1987). Regional variation in Hodgkin's disease incidence by histologic subtype in the US. *Cancer*, **60**, 2841–2847.

GLASER SL. (1994). Reproductive factors in Hodgkin's disease in women: a review. *Am. J. Epidemiol.*, **139**, 237–244.

GRUFFERMAN S AND DELZEL E. (1984). Epidemiology of Hodgkin's disease. *Epidemiol. Rev.*, **6**, 76–106.

GUTENSOHN NM AND SHAPIRO DS. (1982). Social class risk factors among children with Hodgkin's disease. *Int. J. Cancer*, **30**, 433–435.

HUNT JS. (1992). Immunobiology of pregnancy. *Current Opinion in Immunology*, **4**, 591–596.

JARRETT RF. (1993). Viruses and Hodgkin's disease. *Leukaemia*, **2** (suppl. 2), S78–S82.

KRAVDAL O AND HANSEN S. (1993). Hodgkin's disease: The protective effect of childbearing. *Int. J. Cancer*, **55**, 909–914.

MUELLER N, SWANSON GM, HSIEH C AND COLE P. (1987). Tonsillectomy and Hodgkin's disease: results from companion population-based studies. *J. Natl. Cancer Inst.*, **78**, 1–5.

OLSSON H, OLSSON ML AND RANSTAM J. (1990). Late age at first full-term pregnancy as a risk factor for women with malignant lymphoma. *Neoplasma*, **37**, 185–190.

SERRAINO D, FRANCESCHI S, TALAMINI R, BARRA S, NEGRI E, CARBONE A AND LA VECCHIA C. (1991). Socio-economic indicators, infectious diseases and Hodgkin's disease *Int. J. Cancer*, **47**, 352–357.

ZWITTER M AND LEŠNIČAR H. (1986). Hodgkin's disease as a failure of immune regulation: a re-interpretation of the epidemiological findings. *Neoplasma*, **33**, 107–115.